# Medial patellofemoral ligament reconstruction: A review

**DOI:** 10.1097/MD.0000000000028511

**Published:** 2022-01-07

**Authors:** Yong-qiang Zhang, Zhao Zhang, Meng Wu, Ya-dong Zhou, Sheng-lin Tao, Ya-long Yang, Yang Li, Jun-liang Liu, Peng Li, Yun-sheng Teng, Yong-ming Guo

**Affiliations:** Department of Joint Surgery and Sports Medicine, Weapon Industry 521 Hospital, Xi’an, Shaanxi, China.

**Keywords:** femoral attachment, medial patellofemoral ligament (MPFL), patellar dislocation, patellar instability, reconstruction

## Abstract

**Introduction:**

Reconstruction of the medial patellofemoral ligament (MPFL) is an effective surgical method for the treatment of lateral patellar instability. At present, there is not much controversies regarding the femoral attachment, however, the controversies regarding patellar attachment versus attachment, number of graft strands, tension, isometry and so on. The following electronic databases will be searched: PubMed, the Cochrane Library, Embase, Web of Science, Medline. We will consider articles published between database initiation and March 2021. MPFL in the subject heading will be included in the study. Language is limited to English. Research selection, data extraction, and research quality assessment were independently completed by 2 researchers.

**Conclusions:**

MPFL reconstruction is a reliable technique for the treatment of patellofemoral instability. The Schöttle point is still the mainstream method for locating the femoral attachment, the patellar attachment for single-bundle is located at the junction of the proximal one third and the distal two third of the longitudinal axis of the patella. For double-bundles, one is located in the proximal one third of the medial patellar edge and another is in the center of the patellar edge. Meanwhile, the adjustment of graft tension during operation is very important.

## 1 Introduction

Lateral patellar instability is common in sport medicine, which is mainly seen in young people and active patient populations. Greater than two dislocations events lead to a rate of patellar instability greater than 50%.^[1]^ Although there are many factors affecting the stability of the patellofemoral joint, such as increased the tibial-tuberosity to trochlear groove distance (TT–TG distance) distance, trochlear dysplasia, patella altar, patella tilt and so on, medial patellofemoral ligament (MPFL) is considered to be the most important soft tissue structure to restrain lateral patella dislocation.^[2]^ MPFL reconstruction can definitely improve the stability of patellofemoral joint.^[3]^ Therefore, this surgical technique is commonly used in the treatment of lateral patellar instability. At present, there are many controversies about the surgical procedure of MPFL reconstruction. After reviewing a large number of literature, this article may serve to optimize MPFL reconstruction by providing application of guidelines.

## 2 Methods

The systematic review will be performed following the guidelines of the Preferred Reporting Items for Systematic Review and Meta-Analysis Protocols (PRISMA-P) guidelines. This protocol has been registered on INPLASY (registration number: INPLASY202180024: https://inplasy.com/inplasy-2021-8-0024). Ethical approval is unnecessary because this is a literature-based study.

### 2.1 Inclusion criteria

#### 2.1.1 Types of participants

We will consider patients with the operation of MPFL reconstruction irrespective of their sex, age, severity, and disease duration.

#### 2.1.2 Types of interventions

The treatment group using MPFL reconstruction while the control group received treatment with oral medication, physical therapy, or even with no treatment, will be included.

#### 2.1.3 Types of outcomes

The primary outcome of Knee joint pain symptom is visual analog scale (0–10), the ability assessment of daily living activities. Adverse events incidence and knee joint range of motion will be accepted as the secondary outcomes.

### 2.2 Data sources and search methods

#### 2.2.1 Electronic searches

Relevant studies will be searched in the following electronic databases: PubMed, the Cochrane Library, Embase, Web of Science, and Medline databases. We will consider articles published between database initiation and March 2021. In addition, we manually retrieve other resources, including the reference lists of identified publications, conference articles, and gray literature. The following search terms will be used: MPFL; reconstruction; patellar dislocation; patellar instability; femoral attachment, etc. All search terms are included in Table 1, and other searches will be based on these results.

**Table 1:** Search strategy used in PubMed.

Number	Search terms
1	medial patellofemoral ligament
2	MPFL
3	patellofemoral ligament
4	knee ligaments
5	medial support belt
6	Or 1–5
7	MPFL reconstruction
8	reconstruction of the medial patellofemoral ligament
9	lateral patellar instability
10	dislocation of the patella
11	patellar dislocation
12	patellar instability
13	association
14	relation
15	Or 7–14
16	femoral attachment
17	knee accessories
18	knee attachment
19	patella attachment
20	study
21	studies
22	Or 16–21
23	6 and 15 and 22

#### 2.2.2 Searching for other resources

Additionally, the international clinical trials registry platform, dissertation, and gray literature will also be searched to identify systematic reviews related to MPFL reconstruction for Lateral patellar instability. The relevant conference papers, journals will be retrieved manually.

### 2.3 Data collection and analysis

#### 2.3.1 Selection of studies

**Figure 1:**
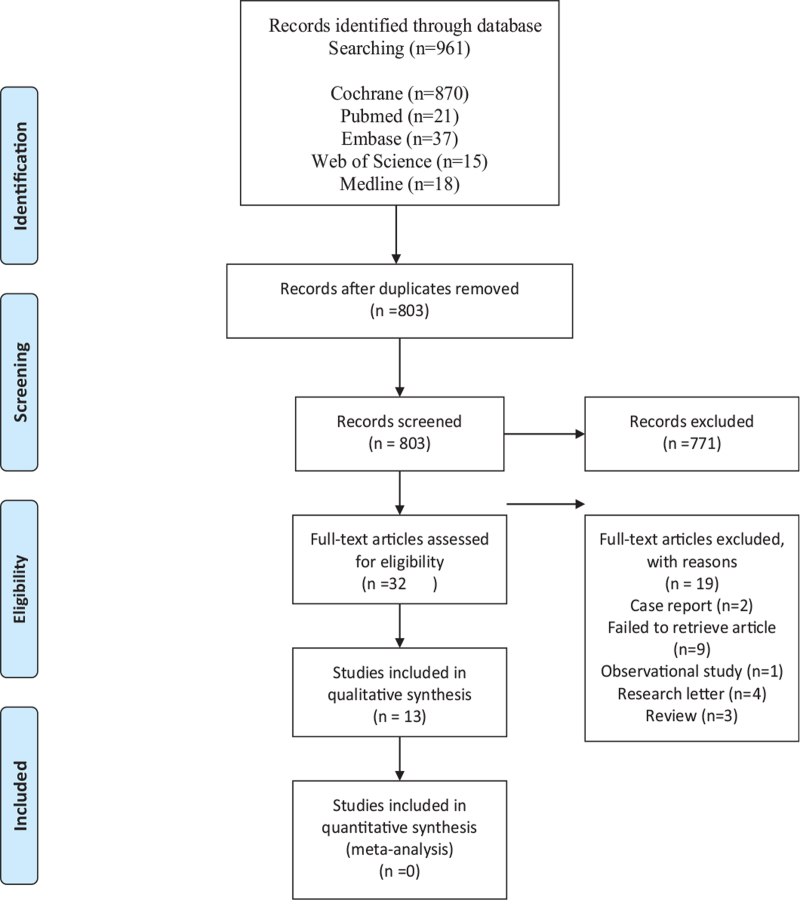
Flowchart of study selection.

Selection of studies. Two researchers will independently discuss and determine research selection process according to the criteria. All literatures will be imported to the endnote X9. We will remove the duplicated data and screen records by title and abstract and the full article. Any study excluded should be labeled on full article. If there is difference in the research choices, we will resolve it by discussing with the third author. Screening study flow diagram is summarized as Figure 1.

#### 2.3.2 Data extraction and management

A standard form will be designed for data collection first. Two researchers will independently extract data of studies and record on the form. For the ambiguity of studies, it will be solved by expert discussion. We will contact authors for more information when necessary. The extracted data contain the first author, publication year, participants characteristics, interventions, duration of treatment, follow-up, outcome assessment, research results, adverse events, and other detailed information. If any details of the article are incomplete, we will contact the appropriate author for more information.

#### 2.3.3 Assessment of risk of bias

Two researchers will independently evaluate the risk and bias using the Cochrane collaboration's tool. These items included in this toll will be evaluated: random sequence generation, allocation concealment, the blinding method for patients, researchers and outcomes assessors, incomplete outcome data, and selective reports. The bias risk for every item will be classed as “low risk of bias,” “high risk of bias,” “unclear risk of bias.”

#### 2.3.4 Measures of treatment effect

For continuous data, a mean difference or standardized mean difference with 95% confidence intervals (CIs) will be applied. For dichotomous outcome data, the risk ratio with 95% Cis will be used to evaluate the treatment effect.

#### 2.3.5 Missing data management

For some articles, if there is incomplete data, we will try to contact the first or corresponding author by email. If the missing data is not available, we will analyze the data acquired.

#### 2.3.6 Assessment of heterogeneity

The research will be performed by Review Manager Version 5.3 software. Heterogeneity will be evaluated by chi-squared test. If I^2^ value is less than 50%, indicating significant heterogeneity statistical results, we will use random effects model. If not, the fixed effects model, standardized mean difference, and corresponding 95% CIs will be applied for further data.

#### 2.3.7 Data synthesis

Data synthesis will be performed using RevMan V.5.4 (The Cochrane Collaboration, London, United Kingdom). The results are expressed as a risk ratio and the standardized or weighted average difference of continuous data. The specific methods were as follows: if the I^2^ test was <50%, the fixed effects model was used for data synthesis. If the I^2^ test was between 50% and 75%, the random-effects model was used for data synthesis. If the I^2^ test is >75%, we will investigate possible reasons from both clinical and methodological perspectives to conduct a subgroup analysis.

If data cannot be synthesized, we provide a descriptive analysis to solve this problem.

#### 2.3.8 Sensitivity analysis

When there are sufficient studies, we will carry out sensitivity analysis to test the robustness of studies according to the quality of method, the sample size, and the selection of missing data. And the fluctuation of results will be observed.

#### 2.3.9 Reporting bias

If there are enough studies include (more than 10), funnel plot will be performed. And the Egger regression and the Begger tests will be calculated to check the asymmetry of funnel plot.

## 3 Results

### 3.1 Study selection and characteristics

**Figure 2:**
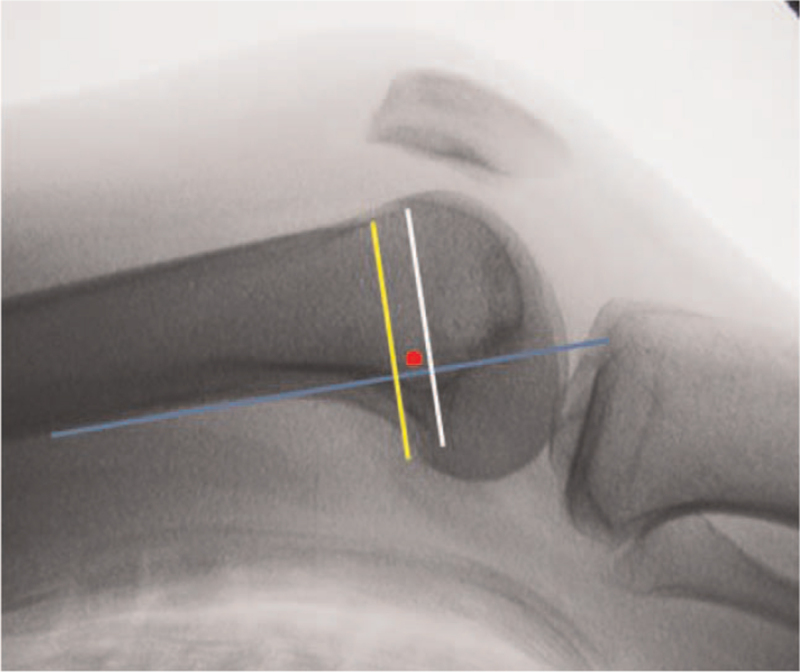
The specific location of the anatomic femoral attachment point on the standard lateral radiograph.

The search strategy identified 961 articles (Fig. 2). After removing duplicates, 803 articles were left to be reviewed. Titles and abstract were retrieved and reviewed for relevance resulting 13 articles. From these studies, we identified 2 case report,9 failed to retrieve article,1observational study,4 research letter,3 review. We decided to exclude these 19 articles and assessed the remaining 9 articles to be reviewed qualitatively. We found no comparable data that can be extracted for quantitative analysis and thus did not perform meta-analysis.

### 3.2 Anatomy and biomechanics

The MPFL was first mentioned in literature by Warren and Marshall^[4]^ who believed that MPFL was a layer of tissue between the medial capsule and the superficial fascial layer of the knee. Some researchers also questioned the existence of MPFL. Reider et al^[5]^ reported that the occurrence rate of MPFL was only 35% in cadavers, while Conlan et al^[6]^ showed that MPFL could not be found in 4 out of 33 knees in specimens. In a systematic anatomical recent retrospective study, the author analyzed literature in the past 20 years and found that MPFL is an anatomical structure that can be easily found in the knee, with an occurrence rate of 99%.^[7]^

The MPFL extends from the patella to the medial condyle with a fan-shaped structure, in fact, patellar insertion has a greater width than the femoral. That is the reason why many investigators suggest double bundles MPFL reconstruction. There are many controversies about MPFL femoral insertion, more of authors think it lies between adductor tubercle and femoral medial epicondyle.^[8]^ A recent study described MPFL femoral attachment in more detail, LaPrade et al showed that the location of the origin was most commonly in the “saddle” between the adductor tubercle and medial epicondyle^[9]^ or within 1 cm distal to the adductor tubercle.^[10]^ In addition, the femoral attachment is spread by decussating fibres that attached to both the adductor tubercle and to the superficial fibres of the medial collateral ligament (MCL), with more direct attachment to the epicondyle.^[11]^ For skeletally immature patients, MPFL femoral insertion is located at 8.5 mm distal to the epiphysis of the medial femoral condyle.^[12]^ Anatomical studies have shown that MPFL femoral attachment is variable, and authors have stressed that the femoral origin should likely be thought of as a “cloud”, rather than a “point”.^[13]^

Some researchers described the proximal MPFL fibers have a wide attachment on patellar and quadriceps tendon. They have referred to this ligament as the medial patellofemoral complex (MPFC) to express more clearly the burgeoning recognition of the diversity of specific insertion sites.^[14]^ Some studies have shown that the proximal MPFL fibers and vastus medialis oblique are connected and interweave each other.^[15,16]^ Tuxoe et al^[17]^ and Mochizuki et al^[18]^ reported that the main tendon with which the MPFL blends its fibers are the vastly intermedius, whereas relations with the vastus medialis oblique are only minimal. Shea et al^[19]^ used a metal needle to mark the MPFL patellar insertion in a specimen study, metal needle position was determined by CT, and the result has shown that MPFL patellar origin is in the upper and medial part of the patella, and the width is 12 mm. Kang et al^[20]^ described two functional bundles based on the patellar insertion. The inferior bundle is a static restraint, and the superior bundle is a dynamic restraint.

Clinically, acute patella dislocation is often accompanied by MPFL tears. There are a few studies on the failure loads of MPFL in biomechanics. Criscenti et al^[21]^ tested the tensile strength of MPFL along the anatomy direction of MPFL, and the results showed that the maximal tensile strength of MPFL is 145N, and the ligament can be stretched up to 9.5 mm before rupturing. He W et al^[22]^ stretched the MPFL from outside to inside and perpendicular to the longitudinal axis of the patella, and the maximum tensile force was 147 N, and the maximum elongated length was 8.4 mm. Mountney et al^[23]^ used a similar method to measure the maximum tensile force of MPFL is 208N, and the ligament can be stretched by 26 mm. Criscenti et al^[21]^ and Smeets et al^[24]^ measured that the maximal length of the MPFL stretched accounted for 24.3% and 22.2% of its own length, respectively. However, these studies are all cadaver or specimen studies and cannot reflect the state of in vivo ligaments. Most of the cadavers and specimens were from older donors, which could not reflect the ligament status of young people, while patellar dislocation often occurs in young people. Oliveira et al^[25]^ showed that MPFL in patients with patellar instability would be longer and thinner compared with asymptomatic people, so the tensile force is weaker and patellar dislocation is more likely to be induced.

Some in vitro studies compared the patellar motion patterns of a healthy knee and MPFL absent knee during knee flexion. It was found that the patella of healthy knee shifted medially during initial flexion of 30°and then shifted laterally in the process of knee flexion of 90°. In contrast, for MPFL-deficient knee, the patella shifted 1 mm to 5 mm laterally when the knee was overextended, and still showed a lateral shift after knee flexion of 30° into the trochlear groove.^[26,27]^ The results suggest that MPFL plays an important role in limiting the lateral patellar dislocation in the early stage of knee flexion. Stephen et al^[28]^ and Sandmeier et al^[29]^ reported that the patella moved laterally during knee flexion, and Sandmeier et al^[29]^ found that the patella shifted laterally in both the healthy knee and the MPFL absent knee, but the degree of lateral displacement of the patella was significantly increased in the MPFL absence knee. They concluded that MPFL was the most important restraint to lateral patellar displacement during knee flexion from 0° to 30°. Sanders et al^[30]^ found that reduced medial traction of the patella after MPFL injury may increase the risk of patellar dislocation, and may also lead to osteochondroma injury and osteoarthritis.

### 3.3 Identification of femoral attachment

**Figure 3:**
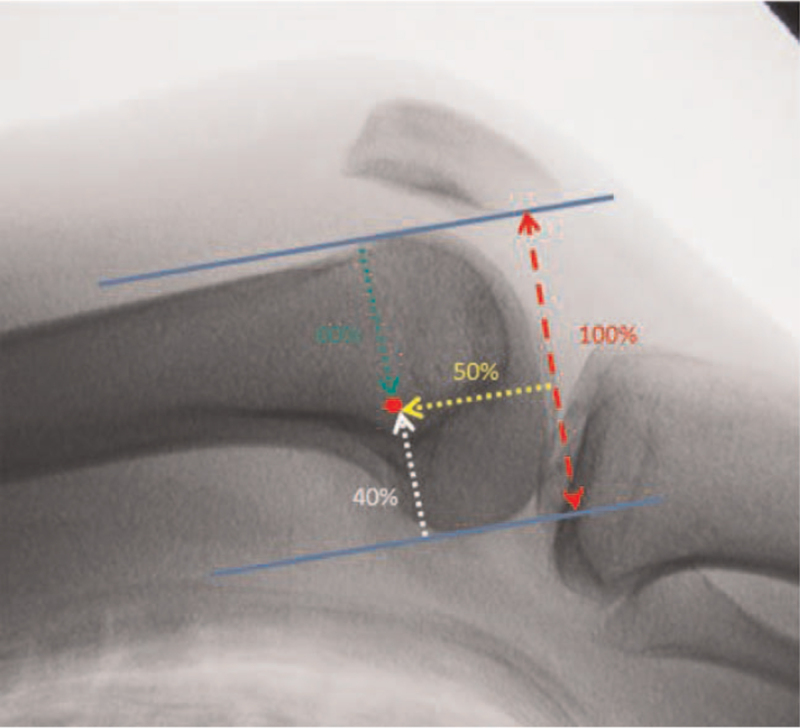
Medial patellofemoral ligament (MPFL) in relation to the size of the medial femoral condyle.

In a cadaveric study, Schöttle et al^[31]^ proposed a method for the first time to locate the MPFL anatomic femoral insertion by relying on radiological markers. They clearly identified the specific location of the anatomic femoral attachment point on the standard lateral radiograph (Fig. 3). It is located 1 mm anterior to the tangent to the posterior femoral cortex, 2.5 mm distal to the perpendicular line traced through the initial part of the medial femoral condyle, and proximal to the perpendicular line traced through the most posterior part of the Blumensaat's line. Schöttle point is widely used in clinical practice. Surgeons can get the MPFL anatomic femoral attachment point according to the standard lateral X-ray of intraoperative fluoroscopy. The limitation of Schöttle point is that it was defined on normal knees. However, most patients with patellar instability have anatomic variations such as trochlear dysplasia or MPFL congenital deficiency. Therefore, there are some limitations in the application of Schöttle point to patients with patellofemoral joint instability. Kaywan Izadpanah et al^[32]^ also confirmed this view. They reported that radiographic landmark-based femoral tunnel placement provides high accuracy in knees with a normal shaped trochlea or mild trochlear dysplasia. However, in patients with severe dysplasia fluoroscopy guided tunnel placement had a low accuracy, exceeding the critical threshold of 5 mm distance to the anatomic MPFL insertion irrespective of the radiographic perspective. Stephen et al^[13]^ proposed that the contour of the posterior femoral condyle could be worn according to the weight-bearing activity of the patient, so the posterior femoral condyle cannot be used as an unchangeable anatomical reference for the location of the femoral insertion point. In order to avoid the limitations of Schöttle's research,^[31]^ Stephen et al^[13]^ used normalized dimensions of the articular geometry and determined the anatomic femoral attachment of the MPFL in relation to the size of the medial femoral condyle: if anterior–posterior size is 100%, then the MPFL attachment is 40% from the posterior, 50% from the distal (Fig. 2). Nevertheless, some researchers still locate the femoral insertion by touching the local anatomic markers of the medial femoral condyle, they believe that the anatomic femoral attachment was located in the “saddle” between adductor tubercle and medial epicondyle.^[33]^ There are also people who identify the adductor tubercle and the medial femoral epicondyle during the operation, and regard the midpoint between them as the MPFL femoral insertion point, and the postoperative results are satisfactory.^[34]^

**Figure 4:**
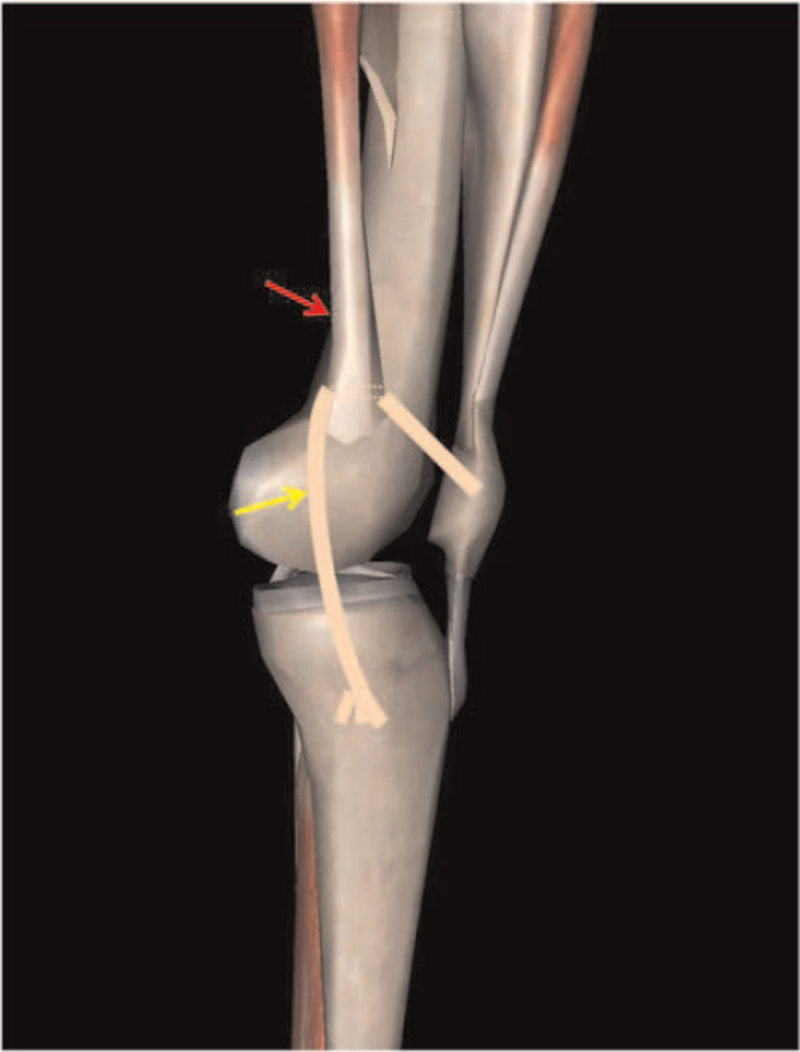
By took autogenous semitendinosus tendon, passed the proximal end of the tendon through the adductor tendon of the adductor tubercle.

**Figure 5:**
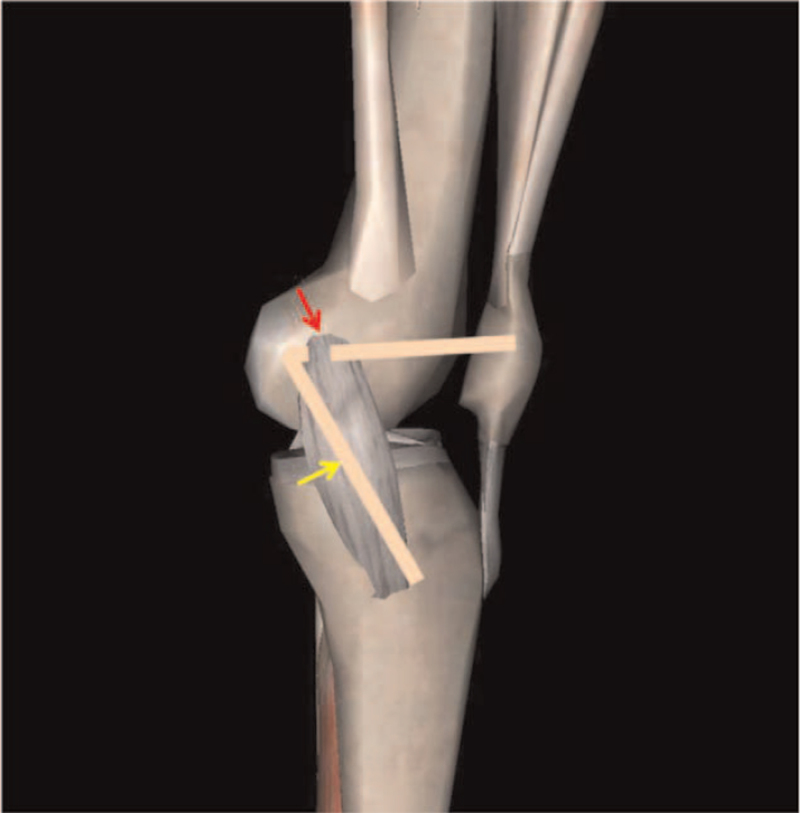
By took autogenous semitendinosus tendon, passed the proximal end of the tendon through the posterior third of the medial collateral ligament (MCL).

Some studies have confirmed that drilling of the MPFL femoral tunnel is safe in a skeletally immature individual.^[35,36]^ However, care should be taken during operation to avoid iatrogenic irreversible damage to the epiphysis. If the intraoperative drilling could not be accomplished safely, the methods reported by Alm et al^[37]^ (Fig. 4) and Deie et al^[38]^ can be used (Fig. 5). Alm et al^[37]^ took autogenous semitendinosus tendon, retained the distal tibial attachment, past the proximal end of the tendon through the adductor tendon of the adductor tubercle, and then fixed the end of the tendon on the patella. They found that 87% of the patients who were operated on using the adductor sling technique gain a stable patella and excellent results in postoperative score. Deie et al^[38]^ used a similar approach, they passed the proximal end of the tendon through the posterior third of the MCL. This method was used to treat recurrent patellar dislocation and habitual patellar dislocation in children with satisfactory clinical results. In addition, the application of anchor fixation at the MPFL femoral insertion can also avoid epiphyseal injury.^[39]^

The correlation between the position of the femoral tunnel and the function of the knee is still controversial. Hopper et al^[40]^ had shown that good clinical results can be obtained as long as the reconstructed femoral insertion is located in the 10 mm of the normal anatomical point. Servien et al^[41]^ and Melegari et al^[42]^ found that there was no correlation between bone tunnel malposition and knee joint function.

### 3.4 Identification of patellar attachment

**Figure 6:**
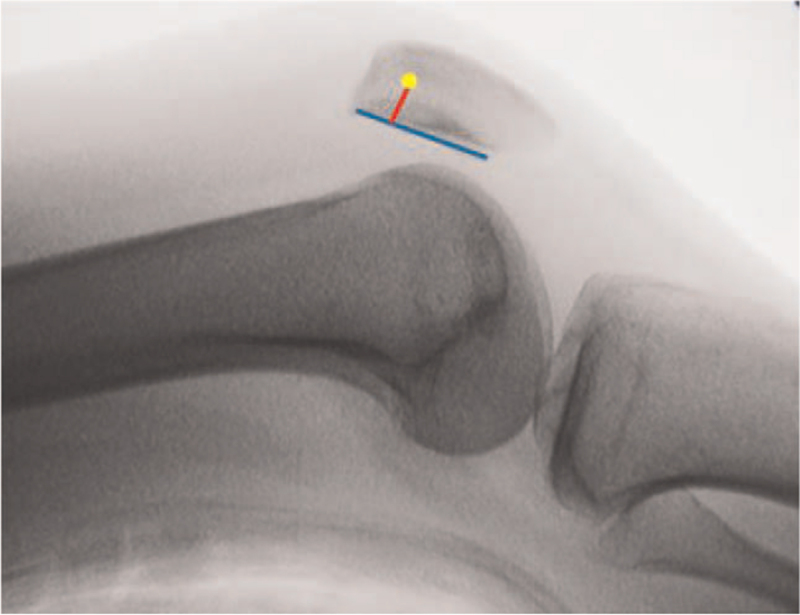
The medial patellofemoral ligament (MPFL) patellar located on the imaging signs.

Barnett et al^[43]^ reported that the MPFL patellar attachment was located by radiographic landmarks. They described that the patellar attachment averaged 7.4 ± 3.5 mm anterior to the posterior patellar cortical line, 5.4 ± 2.6 mm distal to the perpendicular line intersecting the proximal margin of the patellar articular surface (Fig. 6). Furthermore, The MPFL patellar attachment encompasses 33% of the entire length of the patella and is located at the junction of the proximal one third and the distal two third of the longitudinal axis of the patella.^[44]^ However, this localization method is only suitable for single-bundle MPFL reconstruction. Many investigators believed that the patellar double tunnel technique was the closest method to MPFL anatomical reconstruction. The patellar attachment of double-bundle MPFL reconstruction is two points: one from the proximal one third of the medial patellar edge and another from the center of the patella. Schiphouwer et al^[45]^ showed that MPFL reconstruction of patellar double bony tunnel taken the risk of leading to patellar fracture. Considering this factor, some authors used anchors to fix the MPFL patellar insertion. However, some studies have shown that anchor fixation can also cause patellar fracture.^[46]^ There are a variety of fixation methods on the patellar side of MPFL. In comparison, fixation strength of patellar tunnel technique is closest to that of uninjured MPFL.^[34]^

### 3.5 Anatomic and non-anatomic MPFL reconstruction

At present, the controversy about MPFL anatomic and non-anatomic reconstruction is mainly focused on the femoral attachment. Most of the MPFL patellar attachment reconstruction may be non-anatomic reconstruction. Many surgeons supported the anatomic MPFL reconstruction. Burrus et al^[47]^ believed that it is very important to fix the graft on the anatomic insertion point of the femur in MPFL reconstruction. Elias et al^[48]^ analyzed the effect of MPFL reconstruction on the stress and pressure distribution of the patellofemoral joint. They reported that changes in the graft length caused by a technical error at the femoral insertion may increase the stress of the patellofemoral joint and the pressure of the medial patellofemoral cartilage, resulting in overloading of the medial cartilage, further leading to patellofemoral arthritis and pain. Clinical studies have shown that the malposition of femoral attachment in non-anatomical MPFL reconstruction is closely related to postoperative complications.^[49]^ Bollier et al^[50]^ showed in a clinical study that anterior malpositioning of the femoral tunnel can cause overloading of the medial patellofemoral cartilage. Thaunat and Erasmus^[51]^ suggested that a femoral tunnel that is too far proximal may cause graft laxity in extension and graft tension in flexion, which is clinically characterized by anterior knee pain and loss of flexion activity. Moreover, excessive graft tension with knee flexion could stretch the graft and lead to its failure, which may lead to redislocation of the patella. On the contrary, a femoral tunnel that is too distal may lead to graft tension in extension and laxity in flexion. Its clinical manifestation would be an extension lag. Schüttler et al^[52]^ had shown that the widening of the femoral tunnel after MPFL reconstruction is related to the malposition of the femoral attachment. Although many surgeons prefer anatomic MPFL reconstruction, the incidence of non-anatomic reconstruction of the femur insertion is as high as 60%.^[42,53]^ Many studies had also reported that the position of the femoral attachment is not related to the subjective and objective results of postoperative patients, including postoperative motion of knee, pain, apprehension, patellar track, incidence of patellar dislocation and so on.^[42,54,55]^ They showed that anatomic or non-anatomic MPFL reconstruction does not affect the outcome of the operation. Philippe et al^[49]^ reported that although the postoperative complications of MPFL reconstruction are related to the malposition of the femoral tunnel, a malposition does not necessarily lead to poor clinical results. Deie et al^[38]^ took the posterior one third of the MCL attachment as the fixed position for MPFL femoral insertion, and the semitendinosus autograft was fixed on the medial patellar edge after bypassing MCL. This dynamic non-anatomic MPFL reconstruction could also obtain good clinical results without recurrent dislocation.

### 3.6 MPFL isometry

An isometric placement of the MPFL implied that a full range of knee motion can be achieved without evident ligament elongation, thereby allowing the graft length to remain constant throughout the range of motion. Thus, isometry would prevent graft failure due to overstretching. However, many researchers have confirmed that MPFL is anisometric in the full range of knee motion. In an anatomic study, Smirk and Morris^[56]^ found that MPFL isometry only appeared within the 0° to 70° range of motion of the knee. Steensen et al^[57]^ reported a length change of 5.4 mm between the femoral and patellar attachments from 0° to 90° of knee flexion, from 0° to 120°, the length change was 7.2 mm. In a study of double bundle MPFL reconstruction, Victor et al^[58]^ showed that the length of MPFL two bundles varied with the movement of the knee. The proximal bundle was tensioned at 0°, while the distal bundle was tensioned at 30° of the knee flexion. Steensen et al^[57]^ and Stephen et al^[13]^ found that the position of MPFL graft femoral attachment affected its length change, while the position of the patellar attachment had very little effect. In a clinical study, Tateishi et al^[59]^ showed that the location of the MPFL femoral bone tunnel determined the change of the graft length, if there is a significant change in the length of the graft, it would lead to the failure of the MPFL reconstruction.

Thaunat and Erasmus^[51]^ believed that the principle of MPFL reconstruction should be to obtain isometrc ligament at 0° to 30° knee flexion, which can reproduce the isometric nature of the original ligament. They found that the MPFL graft should be tense during the knee extension, lax during knee flexion, and had a change in the length of at least 5 mm from full extension to deep knee flexion, which would prevent the patellar dislocation. In contrast, a recent clinical laboratory study showed that the anatomic MPFL was an anisometric structure with the longest and highest tension in the knee extension, and it became shorter and less tension in the early stage of knee flexion, then the MPFL remained isometric in length with the increased flexion angle of the knee.^[60]^ They concluded that the MPFL anisometry in the early stage of knee flexion is to prevent the lateral patellar dislocation, and the later period is isometric because MPFL is no longer important for maintaining the stability of the patellofemoral joint with the increase of the flexion angle of the knee. Parker et al^[61]^ compared the kinematics of patellofemoral joint between MPFL isometric reconstruction and anatomic reconstruction in a cadaveric study. They showed that isometric reconstruction could not restore the normal kinematic of patellofemoral joint at any knee flexion angle, while anatomic reconstruction could restore normal patellar track at 0° to 28° knee flexion.

### 3.7 MPFL graft tension

In 2007, Bicos et al^[62]^ put forward the idea that MPFL played a “horse rein” role, with high tension only when the patella was in the process of being dislocated. The tension of graft fixation is also very important in MPFL reconstruction. A perfect MPFL reconstruction may fail because the fixation tension is too loose, or it may cause related complications because the fixation is too tight. Thaunat and Erasmus^[51]^ reported on two cases of restricted knee motion after graft over-tightening, one resulted in loss of extension and another in loss of flexion. If the MPFL graft is over-tightened, it can provoke medial patellar subluxation during knee flexion. Given that there is a high prevalence of medial articular lesions in these patients, care must be taken to avoid overloading the medial patellofemoral joint during reconstruction of the MPFL. However, if the MPFL graft is too lax and lacks tension, it can lead to insufficient medial patellar restraint and recurrent lateral patellar instability.^[51]^ Beck et al^[63]^ had shown in a cadaveric study that excessive tension of MPFL can induce an increase in pressure of the medial patellofemoral joint, and they suggest that MPFL should be fixed under a tension of less than 2 N. Philippot et al^[26]^ suggested 10 N graft tension to restore patellofemoral joint mechanics. Stephen et al^[64]^ showed that a graft tensioned to 2 N was sufficient to maintain the stability of the patellofemoral joint, while tensions of 10 N or more caused increased medial contact pressure and medial patellar tilt. If the contralateral patellofemoral joint is stable, the appropriate MPFL graft tension can be adjusted by using the contralateral side as a reference. However, it is necessary to sterilize both the knees simultaneously during operation so as to accurately compare the lateral displacement of the bilateral patella under anesthesia. For patients with bilateral patellofemoral joint instability, the method of Koh and Stewart^[65]^ can be applied. They described that postoperative MPFL graft tension should allow lateral displacement of the patella with 1 cm during the knee extension, or approximately two patellar quadrants of lateral translation with a rigid stop.

Another important question involves the most appropriate knee flexion angle for tensioning the MPFL graft. Reviewing literature, it is controversial, and there is no final conclusion. Thaunat and Erasmus^[66]^ suggested that the MPFL graft should be tightened and fixed in full knee extension. Feller et al^[67]^ fixed the MPFL graft with knee flexion of 20°. Farr and Schepsis^[68]^ fixed the graft at 30° flexion of the knee, resulting in laxity of the MPFL graft during knee flexion and over-tightening in terminal of knee extension. However, Yoo et al^[69]^ reported that the best angle for graft fixation should be 30° knee flexion. LeGrand et al^[70]^ recommended that the graft be fixed between 45° and 60°of knee flexion. Steiner et al^[71]^ fixed the graft between 60° and 90°, because the patella was stable in the trochlear groove when the knee flexion was 60° to 90°.

## 4 Discussion

MPFL reconstruction is a reliable technique for the treatment of patellofemoral instability. However, many details of the operation are controversial. The anatomy and biomechanics of MPFL had been studied deeply. At present, the Schöttle point is still the mainstream method for locating the femoral attachment, although there are some inadequacies in their research. The patellar attachment for single-bundle is located at the junction of the proximal one third and the distal two third of the longitudinal axis of the patella. For double-bundles, one is located in the proximal one third of the medial patellar edge and another is in the center of the patellar edge. Anatomic MPFL reconstruction is closer to physiological MPFL, but better clinical results can also be obtained with non-anatomic reconstruction. The adjustment of graft tension during operation is very important, and overtension should be avoided, because it could provoke overloading on the cartilage of the medial patellofemoral joint, which would lead to cartilage degeneration and patellar tilt, and in severe cases can induce medial patellar dislocation. The question of knee flexion angle for tensioning and fixing the graft in MPFL reconstruction still needs further study in the future.

## Author contributions

**Data curation:** Zhao Zhang, Meng Wu, Ya-dong Zhou.

**Writing – original draft:** Yong-qiang Zhang, Sheng-lin Tao, Ya-long Yang, Yang Li, Jun-liang Liu, Peng Li.

**Writing – review & editing:** Yong-qiang Zhang, Yun-sheng Teng, Yong-ming Guo.

## References

[1] Fithian DC Paxton EW Stone ML. Epidemiology and natural history of acute patellar dislocation. Am J Sports Med 2004;32:1114–21. 15262631 10.1177/0363546503260788

[2] Desio SM Burks RT Bachus KN. Soft tissue restraints to lateral patellar translation in the human knee. Am J Sports Med 1998;26:59–65. 9474403 10.1177/03635465980260012701

[3] Testa EA Camathias C Amsler F. Surgical treatment of patellofemoral instability using trochleoplasty or MPFL reconstruction: a systematic review. Knee Surg Sports Traumatol Arthrosc 2017;25:2309–20. 26187008 10.1007/s00167-015-3698-1

[4] Warren LF Marshall JL. The supporting structures and layers on the medial side of the knee: an anatomical analysis. J Bone Joint Surg 1979;61:56–62. 759437

[5] Reider B Marshall JL Koslin B. The anterior aspect of the knee joint. J Bone Joint Surg 1981;63:351–6. 7204430

[6] Conlan T Garth WP Jr Lemons JE. Evaluation of the medial soft tissue restraints of the extensor mechanism of the knee. J Bone Joint Surg 1993;75:682–93. 8501083 10.2106/00004623-199305000-00007

[7] Placella G Tei M Sebastiani E. Anatomy of the medial patello–femoral ligament: a systematic review of the last 20 years literature. Musculoskelet Surg 2015;99:93–103. 24997630 10.1007/s12306-014-0335-y

[8] Nomura E Inoue M Osada N. Anatomical analysis of the medial patellofemoral ligament of the knee, especially the femoral attachment. Knee Surg Sports Traumatol Arthrosc 2005;13:510–5. 15895206 10.1007/s00167-004-0607-4

[9] LaPrade MD Kallenbach SL Aman ZS. Biomechanical evaluation of the medial stabilizers of the patella. Am J Sports Med 2018;46:1575–82. 29554436 10.1177/0363546518758654

[10] Fujino K Tajima G Yan J. Morphology of the femoral insertion site of the medial patellofemoral ligament. Knee Surg Sports Traumatol Arthrosc 2015;23:998–1003. 24296991 10.1007/s00167-013-2797-0

[11] Aragao JA Reis FP de Vasconcelos DP. Metric measurements and attachment levels of the medial patellofemoral ligament: an anatomical study in cadavers. Clinics 2008;63:541–4. 18719768 10.1590/S1807-59322008000400021 PMC2664133

[12] Farrow LD Alentado VJ Abdulnabi Z. The relationship of the medial patellofemoral ligament attachment to the distal femoral physis. Am J Sports Med 2014;42:2214–8. 25008257 10.1177/0363546514539917

[13] Stephen JM Lumpaopong P Deehan DJ. The medial patellofemoral ligament: location of femoral attachment and length change patterns resulting from anatomic and nonanatomic attachments. Am J Sports Med 2012;40:1871–9. 22729504 10.1177/0363546512449998

[14] Tanaka MJ Voss A Fulkerson JP. The anatomic midpoint of the attachment of the medial patellofemoral complex. J Bone Joint Surg 2016;98:1199–205. 27440568 10.2106/JBJS.15.01182

[15] Andrikoula S. The extensor mechanism of the knee joint: an anatomical study. Knee Surg Sports Traumatol Arthrosc 2006;14:214–20. 16283173 10.1007/s00167-005-0680-3

[16] Baldwin James L. The anatomy of the medial patellofemoral ligament. Am J Sports Med 2009;37:2355–61. 19729366 10.1177/0363546509339909

[17] Tuxoe JI Teir M Winge S. The medial patellofemoral ligament: a dissection study. Knee Surg Sports Traumatol Arthrosc 2002;10:138–40. 12012030 10.1007/s00167-001-0261-z

[18] Mochizuki T. Anatomic study of the attachment of the medial patellofemoral ligament and its characteristic relationships to the vastus intermedius. Knee Surg Sports Traumatol Arthrosc 2013;21:305–10. 22491706 10.1007/s00167-012-1993-7

[19] Shea KG Polousky JD Jacobs JC. The patellar insertion of the medial patellofemoral ligament in children: a cadaveric study. J Pediatr Orthop 2015;35:31–5. 10.1097/BPO.0000000000000399 25633607

[20] Kang HJ Wang F Chen BC. Functional bundles of the medial patellofemoral ligament. Knee Surg Sports Traumatol Arthrosc 2010;18:1511–6. 20232052 10.1007/s00167-010-1090-8

[21] Criscenti G De Maria C Sebastiani E. Material and structural tensile properties of the human medial patello-femoral ligament. J Mech Behav Biomed Mater 2016;54:141–8. 26454357 10.1016/j.jmbbm.2015.09.030

[22] He W Yang Y Liu M. Reconstruction of the medial patellofemoral ligament using hamstring tendon graft with different methods:a biomechanical study. Chinese Med Sci J 2013;28:201–5. 10.1016/s1001-9294(14)60002-x 24382220

[23] Mountney J Senavongse W Amis AA. Tensile strength of the medial patellofemoral ligament before and after repair or reconstruction. J Bone Joint Surg 2005;87:36–40. 15686235

[24] Smeets K Slane J Scheys L. Mechanical analysis of extra-articular knee ligaments, part one: native knee ligaments. Knee 2017;24:949–56. 28784565 10.1016/j.knee.2017.07.013

[25] de Oliveira Victor de Souza Vanessa Cury Ricardo. Medial patellofemoral ligament anatomy: is it a predisposing factor for lateral patellar dislocation? Int Orthop 2014;38:1633–9. 24817023 10.1007/s00264-014-2357-3 PMC4115117

[26] Philippot R Boyer B Testa R. Study of patellar kinematics after reconstruction of the medial patellofemoral ligament. Clin Biomech 2012;27:22–6. 10.1016/j.clinbiomech.2011.08.001 21908083

[27] Stephen JM Kader D Lumpaopong P. Sectioning the medial patellofemoral ligament alters patellofemoral joint kinematics and contact mechanics. J Orthop Res 2013;31:1423–9. 23629829 10.1002/jor.22371

[28] Stephen J Williams A Zaffagnini S. Effect of medial patellofemoral ligament reconstruction method on patellofemoral contact pressures and kinematics. Am J Sports Med 2016;44:1186–94. 26944575 10.1177/0363546516631736

[29] Sandmeier RH Burks RT Bachus KN. The effect of reconstruction of the medial patellofemoral ligament on patellar tracking. Am J Sports Med 2000;28:345–9. 10843125 10.1177/03635465000280031001

[30] Sanders TL Johnson NR Stuart MJ. Patellofemoral arthritis after lateral patellar dislocation: a matched population-based analysis. Am J Sports Med 2017;45:1012–7. 28005405 10.1177/0363546516680604

[31] Schöttle PB Schmeling A Rosenstiel N. Radiographic landmarks for femoral tunnel placement in medial patellofemoral ligament reconstruction. Am J Sports Med 2007;35:801–4. 17267773 10.1177/0363546506296415

[32] Kaywan I Hans M Johanna K. Fluoroscopic guided tunnel placement during medial patellofemoral ligament reconstruction is not accurate in patients with severe trochlear dysplasia. Knee Surg Sports Traumatol Arthrosc 2019;20:2380–4. 10.1007/s00167-019-05413-6 31055609

[33] Wang F Kang HJ Chen BC. Combination of medial patellofemoral ligament reconstruction with vastus medialis advancement for chronic patellar dislocation. Chin Med J 2010;123:3024–9. 21162950

[34] Gao G. Treatment of patellar dislocation with arthroscopic medial patellofemoral ligament reconstruction using gracilis tendon autograft and modified double patellar tunnel technique: minimum 5-year patient-reported outcomes. J Orthop Surg Res 2020;15:25. 31969181 10.1186/s13018-020-1556-4 PMC6977302

[35] Hennrikus W Pylawka T. Patellofemoral instability in skeletally immature athletes. J Bone Joint Surg 2013;95:176–83. 23441343

[36] Vavken P Wimmer MD Camathias C. Treating patella instability in skeletally immature patients. Arthroscopy 2013;29:1410–22. 23714402 10.1016/j.arthro.2013.03.075

[37] Alm L Krause M Mull C. Modified adductor sling technique: a surgical therapy for patellar instability in skeletally immature patients. Knee 2017;24:1282–8. 28867290 10.1016/j.knee.2017.08.051

[38] Deie M Ochi M Sumen Y. Reconstruction of the medial patellofemoral ligament for the treatment of habitual or recurrent dislocation of the patella in children. J Bone Joint Surg 2003;85:887–90. 12931813

[39] Song SY Kim IS Chang HG. Anatomic medial patellofemoral ligament reconstruction using patellar suture anchor fixation for recurrent patellar instability. Knee Surg Sports Traumatol Arthrosc 2014;22:2431–7. 24154711 10.1007/s00167-013-2730-6

[40] Hopper GP Leach WJ Rooney BP. Does degree of trochlear dysplasia and position of femoral tunnel influence outcome after medial patellofemoral ligament reconstruction? Am J Sports Med 2014;42:716–22. 24458241 10.1177/0363546513518413

[41] Servien E Fritsch B Lustig S. In vivo positioning analysis of medial patellofemoral ligament reconstruction. Am J Sports Med 2010;39:134–9. 20929935 10.1177/0363546510381362

[42] Melegari TM Parks BG Matthews LS. Patellofemoral contact area and pressure after medial patellofemoral ligament reconstruction. Am J Sports Med 2008;36:747–52. 18296543 10.1177/0363546508314410

[43] Barnett AJ Howells NR Burston BJ. Radiographic landmarks for tunnel placement in reconstruction of the medial patellofemoral ligament. Knee Surg Sports Traumatol Arthrosc 2012;20:2380–4. 22246545 10.1007/s00167-011-1871-8

[44] Toritsuka Y Amano H Mae T. Dual tunnel medial patellofemoral ligament reconstruction for patients with patellar dislocation using a semitendinosus tendon autograft. Knee 2011;18:214–9. 20684880 10.1016/j.knee.2010.05.007

[45] Schiphouwer L Rood A Tigchelaar S. Complications of medial patellofemoral ligament reconstruction using two transverse patellar tunnels. Knee Surg Sports Traumatol Arthrosc 2016;25:245–50. 27405577 10.1007/s00167-016-4245-4

[46] Dhinsa BS Bhamra JS James C. Patella fracture after medial patellofemoral ligament reconstruction using suture anchors. Knee 2013;20:605–8. 23916510 10.1016/j.knee.2013.05.013

[47] Burrus MT Werner BC Cancienne JM. Correct positioning of the medial patellofemoral ligament: trouble shooting in the operating room. Am J Orthop 2017;46:76–81. 28437491

[48] Elias JJ Cosgarea AJ. Technical errors during medial patellofemoral ligament reconstruction could overload medial patellofemoral cartilage: a computational analysis. Am J Sports Med 2006;34:1478–85. 16685097 10.1177/0363546506287486

[49] Philippe MT Lukas E Linda P. The relationship of femoral tunnel positioning in medial patellofemoral ligament reconstruction on clinical outcome and postoperative complications. Arthroscopy 2018;34:2410–6. 29789249 10.1016/j.arthro.2018.02.046

[50] Bollier M Fulkerson J Cosgarea A. Technical failure of medial patellofemoral ligament reconstruction. Arthroscopy 2011;27:1153–9. 21664791 10.1016/j.arthro.2011.02.014

[51] Thaunat M Erasmus PJ. Management of overtight medial patellofemoral ligament reconstruction. Knee Surg Sports Traumatol Arthrosc 2009;17:480–3. 19132347 10.1007/s00167-008-0702-z

[52] Schüttler KF Hoeger A Heyse TJ. Femoral tunnel widening is associated with tunnel malposition but not with clinical failure after medial patellofemoral ligament reconstruction with a free gracilis tendon graft. Arch Orthop Trauma Surg 2018;138:979–84. 29611007 10.1007/s00402-018-2923-z

[53] McCarthy M Ridley TJ Bollier M. Femoral tunnel placement in medial patellofemoral ligament reconstruction. Iowa Orthop J 2013;33:58–63. 24027462 PMC3748893

[54] Neri T Philippot R Carnesecchi O. Medial patellofemoral ligament reconstruction: clinical and radiographic results in a series of 90 cases. Orthop Traumatol Surg Res 2015;101:65–9. 25530480 10.1016/j.otsr.2014.09.023

[55] Hiemstra LA Kerslake S Lafave M. Medial patellofemoral ligament reconstruction femoral tunnel accuracy: relationship to disease-specific quality of life. Orthop J Sports Med 2017;59:10.1177/2325967116687749 PMC5302095 28210659

[56] Smirk C Morris H. The anatomy and reconstruction of the medial patellofemoral ligament. Knee 2003;10:221–7. 12893143 10.1016/s0968-0160(03)00038-3

[57] Steensen RN Dopirak RM McDonald WG. The anatomy and isometry of the medial patellofemoral ligament: Implications for reconstruction. Am J Sports Med 2004;32:1509–13. 15310579 10.1177/0363546503261505

[58] Victor J Wong P Witvrouw E. How isometric are the medial patellofemoral, superficial medial collateral, and lateral collateral ligaments of the knee? Am J Sports Med 2009;37:2028–36. 19589921 10.1177/0363546509337407

[59] Tateishi T Tsuchiya M Motosugi N. Graft length change and radiographic assessment of femoral drill hole position for medial patellofemoral ligament reconstruction. Knee Surg Sports Traumatol Arthrosc 2011;19:400–7. 20811734 10.1007/s00167-010-1235-9

[60] Kernkamp WA. The Medial patellofemoral ligament is a dynamic and anisometric structure an in vivo study on length changes and isometry. Am J Sports Med 2019;47:1645–53. 31070936 10.1177/0363546519840278

[61] Parker DA Alexander JW Conditt MA. Comparison of isometric and anatomic reconstruction of the medial patellofemoral ligament: a cadaveric study. Orthopedics 2008;31:339–43. 18453169 10.3928/01477447-20080401-28

[62] Bicos J Fulkerson J Amis A. Current concepts review: the medial patellofemoral ligament. Am J Sports Med 2007;35:484–92. 17303819 10.1177/0363546507299237

[63] Beck P Brown NAT Greis PE. Patellofemoral contact pressures and lateral patellar translation after medial patellofemoral ligament reconstruction. Am J Sports Med 2007;35:1557–63. 17435060 10.1177/0363546507300872

[64] Stephen JM Kader D Lumpaopong P. The effect of femoral tunnel position and graft tension on patellar contact mechanics and kinematics after medial patellofemoral ligament reconstruction. Am J Sports Med 2014;42:364–72. 24275861 10.1177/0363546513509230

[65] Koh JL Stewart C. Patellar instability. Clin Sports Med 2014;33:461–76. 24993410 10.1016/j.csm.2014.03.011

[66] Thaunat M Erasmus PJ. The favourable anisometry: an original concept for medial patellofemoral ligament reconstruction. Knee 2007;14:424–8. 17933540 10.1016/j.knee.2007.08.008

[67] Feller JA Richmond AK Wasiak J. Medial patellofemoral ligament reconstruction as an isolated or combined procedure for recurrent patellar instability. Knee Surg Sports Traumatol Arthrosc 2014;22:2470–6. 24928369 10.1007/s00167-014-3132-0

[68] Farr J Schepsis AA. Reconstruction of the medial patellofemoral ligament for recurrent patellar instability. J Knee Surg 2006;19:307–16. 17080654 10.1055/s-0030-1248123

[69] Yoo YS Chang HG Seo YJ. Changes in the length of the medial patellofemoral ligament: an in vivo analysis using 3-dimensional computed tomography. Am J Sports Med 2012;40:2142–8. 22837430 10.1177/0363546512453301

[70] LeGrand AB Greis PE Dobbs RE. MPFL reconstruction. Sports Med Arthrosc 2007;15:72–7. 17505321 10.1097/JSA.0b013e31803bb513

[71] Steiner TM Torga-Spak R Teitge RA. Medial patellofemoral ligament reconstruction in patients with lateral patellar instability and trochlear dysplasia. Am J Sports Med 2006;34:1254–61. 16567459 10.1177/0363546505285584

